# The direction of work flow matters: influence mechanism of task interdependence on employee proactive work behavior

**DOI:** 10.3389/fpsyg.2023.1176862

**Published:** 2023-06-02

**Authors:** Ting Yu, Yanmei Zhao, Zhengtang Zhang

**Affiliations:** ^1^Department of Human Resource Management, School of Business, Nanjing University, Nanjing, China; ^2^Department of Human Resource Management, School of Business, Nanjing Audit University, Nanjing, China

**Keywords:** initiated task interdependence, received task interdependence, task significance, self-esteem, proactive work behavior

## Abstract

Given the increasing uncertainty in today’s environment, how enterprises implement changes to stimulate employee proactive work behavior has become an important practical topic in the human resources field. This study considers work flow direction and refers to the work characteristic and job demand–resource models to explore the influence of task interdependence (initiated and received) on employee proactive work behavior. We interviewed human resource staff and surveyed employees of an internet company headquartered in Jiangsu, China. The empirical results show that initiated task interdependence has a positive impact on employee proactive work behavior, and task significance plays a mediating role between them. Self-esteem does not affect the positive relationship between initiated task interdependence and task significance, nor does it influence the aforementioned mediating effect of task significance. Moreover, received task interdependence has no significant effect on proactive work behavior, and task significance has no significant mediating effect between them. Self-esteem moderates the relationship between received task interdependence and task significance. Specifically, when self-esteem is low, received task interdependence positively predicts task significance, and when self-esteem is high, the received task interdependence–task significance relationship is not significant. Furthermore, self-esteem moderates the mediating effect of task significance between received task interdependence and proactive work behavior. Specifically, when self-esteem is low, task significance plays a mediating role but not when self-esteem is high. Theoretical contributions and managerial implications are discussed.

## 1. Introduction

The importance of improving employee proactive behaviors, which can be defined as self-initiated actions aimed at enhancing the current situation (Parker et al., 2006; Parker and Collins, 2010), through job design has received sustained attention. One of the various studies that have investigated the impact of job design (e.g., Ohly et al., 2006; Parker et al., 2006; Frese et al., 2007; Ohly and Fritz, 2010; Den Hartog and Belschak, 2012) found that task interdependence, which refers to the extent to which a job relies on others and others depend on it to complete their work (Kiggundu, 1981), is positively related to job commitment (Wageman, 1995; Stewart and Barrick, 2000; Van Der Vegt et al., 2000; Van der Vegt et al., 2001), employee proactive work behavior, extra-role behavior, and job satisfaction (Pearce and Gregersen, 1991; Zheng and Bao, 2006; Cleavenger et al., 2007; Kuthyola et al., 2017; Sahu, 2018; Sahu and Pathardikar, 2018; Wu et al., 2018).

Nonetheless, the majority of prior studies have treated task interdependence as a singular construct, overlooking the prospect that distinct types of task interdependence may yield varying effects on employee proactive behaviors. With regard to the direction of workflow, Kiggundu (1981) differentiated task interdependence into two dimensions: initiated task interdependence and received task interdependence. Initiated task interdependence refers to the degree to which a particular job impacts other jobs through the workflow (e.g., seeking information about apartment/house rentals). On the other hand, received task interdependence pertains to the degree to which the work of one job is influenced by one or more other jobs (e.g., accompanying clients to view the apartment/house, Morgeson and Humphrey, 2006). It is important to consider the potential differential effects of different types of task interdependence on employee proactive behaviors. For instance, initiated task interdependence may have a stronger positive effect on such behaviors, as it signals to employees that their work has the potential to affect others and is therefore more meaningful. By failing to differentiate between initiated and received task interdependence, research may risk drawing inaccurate conclusions and overlooking the unique contribution of each type to employee proactive behaviors.

Based on a job design perspective, the aim of this study is to investigate the impact of different types of task interdependence on employee proactive behaviors. Specifically, this research seeks to examine the potential mediating role of task significance and the moderating effect of self-esteem. Initiated task interdependence and received task interdependence occur at different stages of the workflow and have varying degrees of initiative and impact on others’ work. Consequently, employees perceive different levels of work significance, which can lead to varying levels of positive work behaviors. Moreover, individuals with high self-esteem tend to believe in their capability, excellence, and success (Coopersmith, 1967), and may be more inclined to initiate tasks that affect others’ work, rather than merely accepting tasks that are influenced by others’ work. Therefore, this study posits that individuals with high self-esteem are more likely to perceive the significance of initiated task interdependence and the unimportance of received task interdependence, resulting in stronger effects on employee proactive behaviors. Conversely, individuals with low self-esteem may not exhibit this differentiation due to their self-perception of being less successful and outstanding. As such, the distinction between initiated and received task interdependence may not have a significant impact on their perception of work significance.

This paper contributes to the literature on task interdependence and proactive behaviors in the following three aspects. Firstly, by drawing on the job design perspective, our study theorizes and examines the impact of task interdependence on proactive behaviors. This approach addresses the need to consider job characteristics in relation to employee proactive work behavior and the changing effects of these characteristics over time (Grant and Ashford, 2008). Moreover, by focusing on employee proactive work behavior, this study expands research on positive psychology, which is in line with the current development trend of organizational psychology (Carpintero, 2017).

Secondly, this study aims to uncover the mechanism of task interdependence on employee proactive behaviors by examining the mediating effect of task significance and moderating effect of self-esteem. To comprehensively understand the relationship between task interdependence and employee proactive behaviors, researchers must delve deeper into the underlying mechanisms of these relationships (Grant and Ashford, 2008). As such, our mediating model, moderating model, and moderated mediation model could, to certain extent, make contributions to the task characteristic and employee proactive behavior literature by providing a more comprehensive understanding of how job design can promote employee proactive behavior.

Thirdly, previous studies on task interdependence have often treated it as a single construct, neglecting the potential differences among various types of task interdependence. In contrast, our study tests the differential effects of initiated and received task interdependence, which allows us to explore the nuanced differences and broaden our understanding of the nature of task interdependence.

## 2. Theory and hypotheses

### 2.1. Task interdependence

The most elaborate and widely accepted job characteristic theory earlier on was proposed by Hackman and Oldham (1976), which constructs a model of five core job characteristics: skill variety, task identity, task significance, autonomy, and job feedback. The model holds that these five core job characteristics prompt three psychological states (experience of the work’s meaningfulness, experience of responsibility for the outcomes, and knowledge of the work activities’ actual results) which, in turn, lead to some beneficial personal and work outcomes.

Kiggundu (1981, 1983) developed the concept of task interdependence and integrated it in the theory of job design by Hackman and Oldham (1976). He defined task interdependence as the interrelation and mutual influence between job positions, in which the performance of one job position depends on the successful performance of another job position. At present, collaborative environments in enterprises are becoming more prevalent, and task interdependence has become an important variable affecting employees’ psychological feelings and behaviors at work (Grant and Parker, 2009).

Some empirical studies have found that task interdependence affects the behavior and attitude of employees. Most of these studies explored the relationship between overall interdependence (integrating task interdependence and output interdependence) and team performance at the team level. For example, Wageman (1995) showed a U-shaped relationship between overall interdependence and team performance, and teams with higher task interdependence had better communication and more frequent helping behaviors and information sharing. Stewart and Barrick (2000) took production teams as the research object and found a curved relationship between interdependence and team performance, which was regulated by task types. Specifically, for teams with conceptual (behavioral) tasks, the relationship between interdependence and team performance was U-shaped (inverted U-shaped). The work of Van Der Vegt et al. (2000) on technical consultant teams showed a positive correlation between task interdependence and individual job satisfaction, team satisfaction, job commitment, and team commitment, and this positive correlation was regulated by output interdependence. The study of Van der Vegt et al. (2001) on teaching teams and engineering teams showed a positive correlation between team-level task interdependence and team members’ work satisfaction, and likewise between intra-team job interdependence and work satisfaction but only when team goal interdependence was high.

There are relatively few studies at the individual level regarding the influence of job task interdependence on individuals. For example, Pearce and Gregersen (1991) found that task interdependence influences employees’ extra-role behaviors (e.g., helping behavior) by influencing their perception of responsibility to others. Cleavenger et al. (2007) found that task interdependence better promoted employee help-seeking behavior in environments that support helping behavior. Sahu and Pathardikar (2018) studied a manufacturing enterprise in India and found that employee role ambiguity, work interdependence, and trust among colleagues could predict employee job satisfaction. Kuthyola et al. (2017), based on a survey of 300 software personnel, found that task interdependence can improve project performance through the mediating mechanisms of collaboration, cohesion, and learning. Sahu and Pathardikar (2018) investigated a sample of 635 middle- and lower-level managers working in insurance companies, and the results showed that leadership empowerment and task interdependence were positively correlated with employee proactive behaviors and negatively correlated with employee counterproductive behaviors. However, other studies have shown no relationship between perceived task interdependence and professional emotional commitment in Chinese public accounting firms (Zheng and Bao, 2006).

### 2.2. Initiated task interdependence, received task interdependence, and proactive work behavior

The preceding literature review reveals that most of the existing studies take task interdependence as an overall variable. However, the interdependence characteristics of different working positions actually vary. Based on the direction of work flow, Kiggundu (1981) defined and distinguished task interdependence into two dimensions: initiated task interdependence and received task interdependence. Initiated task interdependence is the extent of job task flow from a specific position to other positions; the success of the latter depends on the successful completion of the job initiation position. Received task interdependence refers to the extent to which the work of one position is affected by one or more other jobs.

According to Kiggundu (1981), initiated task interdependence is positively related to work outcomes such as internal work motivation, work satisfaction, growth satisfaction, and quality performance. Employees with a high degree of initiated task interdependence would have a high sense of responsibility for others who accept their tasks, thus improving employees’ intrinsic work motivation, job satisfaction, and performance. Lawler et al. (1968) also argued that managers who initiate interdependent behaviors themselves have greater satisfaction and positive emotions.

However, received task interdependence may have the opposite effect on employees. Thompson (2017) mentioned that received task interdependence reduces employees’ autonomy, thereby lowering their intrinsic motivation and job satisfaction. Trist and Bamforth (1951) found that miners focusing on received task interdependence refused to accept responsibility for the output and had more “self-compensating” absenteeism and turnover than others.

The aforementioned studies reveal that even for job positions characterized by task interdependence, different work flows vary in their impacts on the attitude, behavior, and work outcomes of employees. However, these views lack the support of concrete empirical analysis. Therefore, it is necessary to distinguish the influence of different task interdependence dimensions on employee behavior by considering the direction of work flow and conducting the corresponding empirical tests.

The job demand–resource theory (Bakker and Demerouti, 2007) divides working conditions into two categories: job resources and job requirements. Job resources refer to job features that help in achieving work objectives, reduce job requirements and related costs, promote personal growth and development, increase employees’ work involvement and create positive emotions (Fredrickson, 1998), thus stimulating people to explore new things, improve their ability to adapt to changes, and become more proactive (Hakanen et al., 2008).

Initiated task interdependence is a job resource because the employee who mainly initiates tasks takes up more resources that other people’s work requires, has higher autonomy for work than the employee who mainly receives tasks, and has greater control over the work. Therefore, an initiated task-oriented employee is more likely to generate proactive work behaviors at work.

Job requirements refer to the continuous physical, mental, or skill inputs required at work, which consume employees’ physical and mental resources as energy is expended, possibly leading to stress, burnout, and emotional exhaustion. Received task interdependence means always being influenced by others at work. This is an environmental handicap for an employee and is likely to be perceived by the employee as a threat to his or her goals. Therefore, received task interdependence is considered an obstacle-type job requirement. In addition, employees who mainly receive tasks need to rely on others to complete the work, which reduces the sense of work autonomy and control. Cognitive evaluation theory holds that when the external environment makes an individual feel controlled or incapable, his or her internal motivation will be reduced. All these hinder employees from generating spontaneous change-oriented proactive work behaviors such as innovation, change initiative, voice behavior, and so on. Thus, we propose.

*H_1*a*_*: Initiated task interdependence is positively related to employee proactive work behavior.

*H_1*b*_*: Received task interdependence is negatively related to employee proactive work behavior.

### 2.3. The mediating role of task significance

Task significance refers to the extent to which an individual’s work affects the work and life of others, regardless of whether the impact is on the organization or the external environment (Hackman and Oldham, 1976). For example, the task significance of an ambulance driver is greater than that of a cashier, because the former’s job involves the life and safety of others. Social information processing researchers define task significance as a kind of subjective judgment on work formed by employees in interpersonal communication (Griffin, 1983).

Task interdependence plays a key role in forming employees’ perception of task significance (Wrzesniewski et al., 2003). In addition, different dimensions of task interdependence (initiated or received) vary in their influence on task significance depending on the direction of work flow. Compared with employees who mainly receive tasks, those who mainly initiate tasks feel that their work has an impact on the work of others and even affect the achievement of the whole team’s goal, and they are more likely to think that their work is more important. On the contrary, for employees who mainly receive tasks, the work result is influenced by others, and such employees may feel that they are just passively performing tasks. Compared with employees who initiate tasks, employees in this kind of position feel that their work is not important, because their work is more influenced by others rather than having an impact on others.

When employees find their work important and meaningful, they become more concerned about the quality and effectiveness of their work. For example, Grant (2008) found that task significance increased employees’ perception of social influence and social value, leading them to become more active in seeking ways to perform their jobs, such as active feedback-seeking behavior, innovative ways to solve problems, and active learning to improve their work ability. By contrast, when employees think that their work is not important or meaningful, or their good or bad work does not have a great impact on the life and work of others, their internal work motivation will be reduced, and they will not pay attention to their work, nor will they take the initiative to engage in proactive behavior to improve their work performance. Hence, we propose.

*H_2*a*_*: Task significance plays a mediating role in the influence of initiated task interdependence on employee proactive work behavior.

*H_2*b*_*: Task significance plays a mediating role in the influence of received task interdependence on employee proactive work behavior.

### 2.4. The moderating role of self-esteem

Self-esteem refers to an individual’s self-recognition and the extent to which a person thinks he is “capable, outstanding, successful, and valuable” (Coopersmith, 1967). It plays an important role in predicting employee attitudes and behaviors (Pierce and Gardner, 2004; Bowling et al., 2010). Self-verification theory holds that individuals have a strong need for consistent self-recognition such that they are more willing to engage in activities that are consistent with their self-perception. Individuals with high self-esteem have been found to have a higher sense of self-efficacy and significance, and a higher expectation of success. Therefore, a high self-esteem may motivate individuals to set challenging career goals and be proactive to achieve these goals, to substantiate their high self-esteem. Regarding the direction of work flow, if a person has an initiated task-oriented job position, this indicates that he/she has greater ability to influence the work of others. Individuals with high self-esteem believe that this kind of job position is consistent with their higher self-perception; thus, they feel valued by the company and that their work tasks are important. Meanwhile, initiated task interdependence may bring burden to individuals with low self-esteem; hence, they may adopt an avoidance attitude to deal with it. Therefore, we propose.

*H_3*a*_*: The relationship between initiated task interdependence and task significance is moderated by self-esteem. Compared with individuals with low self-esteem, the positive relationship between initiated task interdependence and task significance is stronger for individuals with high self-esteem.

Similarly, work that is focused on receiving tasks is inconsistent with the self-cognition of individuals with high self-esteem, because employees who focus on receiving tasks are more passive at work; their work results are greatly influenced by others, and they have less autonomy at work. Therefore, in such a situation, individuals with high self-esteem would think that the company does not pay attention to their ability and that their work tasks are unimportant. By contrast, a received task-oriented job position is more consistent with the self-cognition of individuals with low self-esteem.

Duffy et al. (2000) claimed that individuals with low self-esteem tend to conform to the expectations of others and be particularly dependent upon the positive evaluation of themselves by others. They are more adapted to passive job positions such as those under received task interdependence conditions. Moreover, individuals with low self-esteem are more self-regulating and tend to do what others expect under negative situations (Duffy et al., 2000). Received task interdependence is a job requirement for employees that can be understood as a negative situation. Individuals with low self-esteem in such a situation may think that their work is important and suitable, and consequently work hard to obtain recognition from others. Thus, we propose.

*H_3*b*_*: The relationship between received task interdependence and task significance is moderated by self-esteem. For individuals with low self-esteem, received task interdependence has a positive impact on their perception of task significance, whereas for individuals with high self-esteem, received task interdependence has a negative impact on their perception of task significance.

Because self-esteem moderates the relationship between initiated/received task interdependence and task significance, and it was previously assumed that task significance plays a mediating role between initiated/received task interdependence and proactive work behavior, we hypothesize that self-esteem moderates the mediating effect of task significance on this relationship. Specifically, individuals with high self-esteem think that they have strong ability and competence, which are valuable traits for the enterprise. If they mainly hold initiated task-interdependent job positions, they would feel that their ability is better applied and their value can be better recognized, which is consistent with their higher self-cognition. Thus, they would also feel that their work is highly important. If they are entrusted with important tasks, they would be more positive about their work, thus generating more proactive work behaviors to maintain their high self-esteem level. Meanwhile, individuals with low self-esteem are used to taking an avoidance attitude when facing situations that are inconsistent with their lower self-cognition. Therefore, we propose.

*H_4*a*_*: The mediating effect of task significance between initiated task interdependence and proactive work behavior is moderated by self-esteem. For individuals with high self-esteem, task significance plays a stronger mediating role between initiated task interdependence and proactive work behavior.

Furthermore, if the job position of an individual with high self-esteem is more focused on receiving tasks, a relatively passive work would make this individual think that his/her ability is underestimated and consequently tend to see the work as unimportant, which is inconsistent with his/her higher self-cognition; thus, he/she would be unwilling to engage in proactive work behaviors. However, for an individual with low self-esteem, received task interdependence is more consistent with his/her lower self-cognition; thus, he/she is more likely to change his/her avoidant behaviors to obtain the support of others when working in situations of high conflict and high interdependence. By perceiving the work tasks as important, the individual generates more proactive work behaviors. Thus, we propose.

*H_4*b*_*: The mediating effect of task significant between received task interdependence and proactive work behavior is moderated by self-esteem. For individuals with low self-esteem, task significance plays a mediating role between received task interdependence and proactive work behavior. For individuals with high self-esteem, task significance does not mediate between received task interdependence and proactive work behavior.

## 3. Materials and methods

### 3.1. Sample and procedures

**FIGURE 1:**
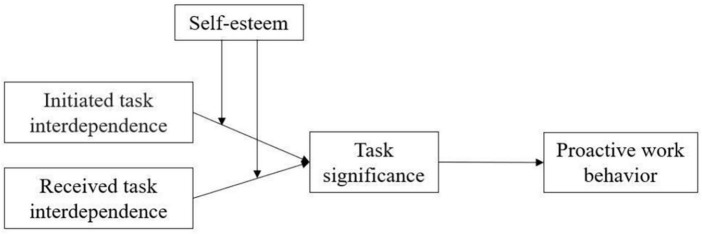
Theoretical model.

To test our theoretical model (see Figure 1), data were collected from employees in a national real estate company in southern China, which specializes in the sale and rental of residential properties. The employees’ workflow encompasses multiple tasks, including collecting information on the housing market, maintaining and updating housing information, guiding customers in viewing apartments and houses, and signing rental or sales contracts. All tasks involve both initiated task interdependence, such as collecting information on the housing market, and received task interdependence, such as guiding customers to view apartments and houses, rendering the company an appropriate sample for this research.

Prior to conducting the survey, we obtained formal permission from the chairman or managing director of the company. Subsequently, the company provided us with employee rosters, and all employees on the roster were invited to participate in the survey through both verbal and written communication (i.e., questionnaire guidelines). Participation in the survey was completely voluntary. In addition, informed consent was obtained from employees who voluntarily chose to participate, granting the researchers permission to access their demographic information through the company’s human resource department. To ensure the questionnaire’s situational applicability, the researchers conducted several interviews with the company’s human resource department and employees, and made appropriate adjustments to some of the questions on the scale. Following this, the researchers administered the survey in accordance with the company’s annual questionnaire.

The data were collected on three separate occasions over the course of a month. Demographic information, including gender, education, and position, was obtained from the company’s HR department. During the first time point (t1), employees were asked to rate their own levels of initiated and received task interdependence as well as their self-esteem. At the second time point (t2), participants rated the level of task significance associated with their work tasks. Finally, at the third time point (t3), employees were invited to rate their proactive work behavior. The current study employed a sample of 1,521 employees in t1, from which 1,463 completed questionnaires were obtained, indicating a response rate of 96.19%. In t2, questionnaires were distributed among all the participants who had filled out the questionnaire in t1, and 1,391 valid responses were received, reflecting a response rate of 95.08%. In t3, questionnaires were distributed among all the participants who had completed the questionnaire in t2, and 1,330 valid responses were received, representing a response rate of 95.61%. In total, the response rate of subordinates was 87.44%. The sample consisted of 53.2% women, and 63.3% had attained an undergraduate degree or above. In terms of their position, 85.56% were basic staff, and the remaining participants were in managerial positions such as department managers, among others.

### 3.2. Measures

To ensure congruence of the Chinese version with the English version of the scales, we followed the widely used translation and back-translation method (Brislin, 1970). All variables in this study were measured with Likert 5-point scale (1 = very strongly disagree 5 = very strongly disagree).

#### 3.2.1. Initiated task interdependence

Initiated task interdependence was measured with a three-item scale developed by Morgeson and Humphrey (2006). The items were “The job requires me to accomplish my job before others complete their job,” “Other jobs depend directly on my job” and “Unless my job gets done, other jobs cannot be completed” (α = 0.73).

#### 3.2.2. Received task interdependence

Received task interdependence was measured with a three-item scale developed by Kiggundu (1983). The items were “The job activities are greatly affected by the work of other people,” “The job depends on the work of many different people for its completion” and “My job cannot be done unless others do their work” (α = 0.83).

#### 3.2.3. Task significance

Task significance was measured using four items developed by Morgeson and Humphrey (2006). The items were “The results of my work are likely to significantly affect the lives of other people,” “The job itself is very significant and important in the broader scheme of things,” “The job has a large impact on people outside the organization” and “The work performed on the job has a significant impact on people outside the organization” (α = 0.76).

#### 3.2.4. Proactive work behavior

Proactive work behavior was measured using thirteen items developed by Parker and Collins (2010), which comprised four dimensions: taking charge (three items), voice (four items), individual innovation (three items), and feedback seeking (three items) (α = 0.88).

#### 3.2.5. Self-esteem

Participants reported their self-esteem using the scale developed by Rosenberg (2015). Sample item is “I have a positive attitude toward myself” (α = 0.78).

#### 3.2.6. Control variables

We controlled for several variables, including gender, education, and position. In the statistical analysis, the variables of education level, gender, and position were treated as dummy variables.

## 4. Results

Using Mplus 7, we conducted a confirmatory factor analysis on the five latent variables, namely, initiated task interdependence, received task interdependence, task significance, self-esteem, and proactive work behavior, with the aim of establishing their discriminant validity. The predicted five-factor solution exhibited adequate fit with the data (χ^2^/df = 4.69, RMSEA = 0.05, CFI = 0.92, TLI = 0.91, SRMR = 0.06). A comparison was delivered between the hypothesized five-factor model and an alternative four-factor model in which the two task interdependence variables (i.e., initiated task interdependence and received task interdependence) were combined into one factor. The results indicated that the five-factor model achieved better fit with the data than the four-factor model (χ^2^/df = 7.22, RMSEA = 0.07, CFI = 0.86, TLI = 0.84, SRMR = 0.07). Additionally, the five-factor model is found to be more suitable than a three-factor model that combined the task interdependence variables as one factor, with self-esteem as a factor, self-esteem as another factor, and the mediator (task significance) and dependent variable (proactive work behavior) as a third factor (χ^2^/df = 7.42, RMSEA = 0.07, CFI = 0.85, TLI = 0.84, SRMR = 0.08), a two-factor model that combined the task interdependence variables and self-esteem as one factor and grouped the other variables as the second factor (χ^2^/df = 10.48, RMSEA = 0.08, CFI = 0.78, TLI = 0.76, SRMR = 0.12), and a single-factor model (χ^2^/df = 6194.85/299, RMSEA = 0.12, CFI = 0.54, TLI = 0.50, SRMR = 0.11). The results indicated the five-factor model provided the best fit compared to the other alternative models, thus supporting the discriminant validity of the variables.

Table 1 displays the means, standard deviations, correlations, and reliability values of all variables. The results showed no relation between received task interdependence and proactive work behavior. However, as expected, initiated task interdependence was positively associated with proactive work behavior (*r* = 0.204, *p* < 0.001) and task significance (*r* = 0.406, *p* < 0.001). Received task interdependence was positively associated with task significance (*r* = 0.352, *p* < 0.001), and self-esteem was positively associated with task significance (*r* = 0.265, *p* < 0.001), and proactive work behavior (*r* = 0.457, *p* < 0.001).

**TABLE 1:** Descriptive statistics and correlations^a^.

Variables	M	SD	1	2	3	4	5	6
1. Gender^b^	0.47	0.826	–					
2. Initiated task interdependence	3.12	0.718	−0.002	–				
3. Received task interdependence	2.67	0.776	−0.008	0.423***	–			
4. Self-esteem	3.91	0.428	−0.027	0.103**	−0.028	–		
5. Task significance	3.54	0.673	0.033	0.406***	0.173***	0.265***	–	
6. Proactive work behavior	3.41	0.478	0.063*	0.204***	0.052	0.457***	0.352***	_

*N* = 1,330.

*p < 0.05; **p < 0.01; ***p < 0.001.

^a^The position and education of control variables are classified variables, which are not included in this table.

^b^1 = male; 2 = female.

As showed in Table 2, only the control variables were added to Model 1, and two independent variables (initiated task interdependence and received task interdependence) were added to Model 2. The result of Model 2 showed that initiated task interdependence positively affected proactive work behavior (β = 0.13, *p* < 0.001), thus supporting H_1*a*_. Meanwhile, the results of M2 also showed that received task interdependence had no significant influence on proactive work behavior; hence, H_1*b*_ is not supported.

**TABLE 2:** Results of hierarchical regression analyses.

Variables	Proactive work behavior			Task significance			
	Model 1	Model 2	Model 3	Model 4	Model 5	Model 6	Model 7
Gender	0.12***	0.11***	0.08***	0.17***	0.15***	0.16***	0.16***
Position1	0.31***	0.28***	0.25***	0.25***	0.17**	0.14**	0.13*
Position2	0.28***	0.27***	0.24***	0.17	0.13	0.12	0.11
Education1	0.11	0.11	0.05	0.26*	0.27*	0.26*	0.26*
Education2	0.23	0.01	0	0.10**	0.06	0.07*	0.07*
Initiated task interdependence		0.13***	0.05**		0.36***	0.35***	0.11
Received task interdependence		−0.02	−0.02		0.004	0.02	0.03
Task significance			0.21***			0.36***	0.37***
Initiated task interdependence × Self-esteem							0.06
Received task interdependence × Self-esteem							−0.12*
*R* ^2^	0.069	0.101	0.173	0.036	0.186	0.238	0.241
Δ*R*^2^	0.069***	0.032***	0.072***	0.036***	0.15***	0.052***	0.003

*N* = 1,330, *p < 0.05; **p < 0.01; ***p < 0.001.

H2a and H2b states the mediating effect of task significance. The results were illustrated in Table 2, Model 2 showed that initiated task interdependence positively affected proactive work behavior (β = 0.13, *p* < 0.001), while Model 5 showed that initiated task interdependence positively affected task significant (β = 0.36, *p* < 0.001). Furthermore, in M3, the independent and mediating variables were simultaneously used to predict the dependent variable. Task significance still had a positive effect on proactive work behavior (β = 0.21, *p* < 0.001), and initiated task interdependence had a decreased but significant effect on proactive work behavior (β = 0.05, *p* < 0.01). According to these results, task significance partially mediated the positive relationship between initiated task interdependence and proactive work behavior, thus supporting H_2*a*_. Meanwhile, the results of Model 2, Model 3, and Model 5 do not support H_2*b*_.

Next, this study examined the mediating role of task significance with a 95% confidence interval by using the bootstrapping method. The 95% confidence interval of the indirect effect of task significance was [0.060, 0.097], which did not contain 0, meaning that the indirect effect was significant; therefore, H_2*a*_ is verified. Moreover, when the initiated task interdependence and task significance were both included in the regression model, the influence of initiated task interdependence on proactive work behavior remained significant, and the CI was [0.005, 0.076]. Therefore, task significance played a partial mediating role between initiated task interdependence and proactive work behavior. We then tested the mediating role of task significance in the influence of received task interdependence on proactive work behavior. The results showed that the CI of the indirect effect of task significance was [−0.037, 0.025], which contained 0, meaning that the indirect effect was not significant; hence, H_2*b*_ is not verified.

In Table 2, only the control variables were inputted in Model 4. Two independent variables (initiated task interdependence and received task interdependence), a moderating variable (self-esteem), and the interaction between the independent and moderating variables were then in turn inputted in Model 5, Model 6, and Model 7. The results of Model 7 showed that after controlling for the influence of initiated task interdependence, received task interdependence, and self-esteem on task significance, the interaction term between received task interdependence and self-esteem had a significant negative effect on task significance (β = −0.12, *p* < 0.05), thus confirming H_3b_. Meanwhile, the interaction term between initiated task interdependence and self-esteem had no significant effect on task significance; hence, H_3a_ was not supported by the data.

**FIGURE 2:**
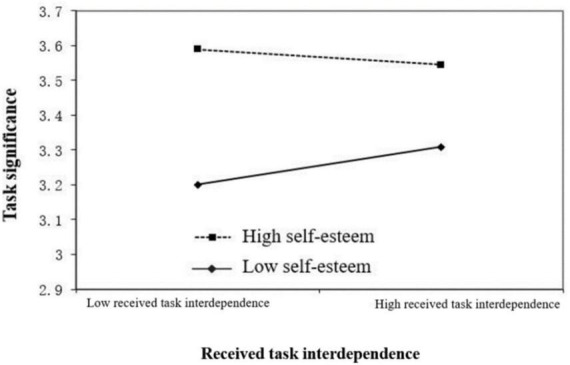
Interaction of self-esteem and received task interdependence predicting task significance.

Aiken et al. (1991) suggested that when the moderating variable is the mean plus one standard deviation and minus one standard deviation, the moderating effect diagram can be drawn. The results showed that when employees’ self-esteem was high, the negative relationship between received task interdependence and task significance was not significant. When employees’ self-esteem was low, received task interdependence and task significance were positively correlated (see Figure 2). This study also conducted a simple effect test to determine whether the slope is significantly non-0 when the adjustment variables are set to high and low values. For individuals with high self-esteem, the simple slope was −0.03, which is not significant and not 0. For individuals with low self-esteem, the simple slope was 0.07, which is significantly not 0 at the level of 5%, indicating that received task interdependence positively affected task significance. Therefore, H_3b_ is supported.

According to the results (see Table 3), the mediating effect between initiated task interdependence and proactive work behavior was significant when self-esteem was high [indirect effect = 0.02, 95%CI (0.013, 0.027)]. When self-esteem was low, this mediating effect remained significant [indirect effect = 0.015, 95%CI (0.010, 0.023)]. The mediating effect difference between the high and low self-esteem conditions was 0.004, and the 95% CI was (−0.003, 0.012), containing 0, thus revealing no significant difference. This means that initiated task interdependence stimulated task significance and similarly brought about proactive work behaviors from both individuals with high or low self-esteem (the mediating effect of task significance was significant), but the mediating effect of task significance was not moderated by self-esteem (the mediating effect of initiated task interdependence was not significant). Thus, H_4a_ is not supported by the data.

**TABLE 3:** Results of moderated mediation.

Moderator	Initiated task interdependence		Received task interdependence	
	Indirect effect	95%CI	Indirect effect	95%
High self-esteem	0.02	(0.013, 0.027)	−0.002	(−0.006, 0.001)
Low self-esteem	0.015	(0.010, 0.023)	0.006	(0.002, 0.011)
Difference	0.004	(−0.003, 0.012)	−0.008	(−0.015, −0.002)

*N* = 1,330; CI = confidence interval.

As showed in Table 3, the mediating effect between received task interdependence and proactive work behavior was not significant for employees with high self-esteem [indirect effect = −0.002, 95%IC (−0.006, 0.001)], but for those with low self-esteem, this mediating effect was significant [indirect effect = 0.006, 95%IC (0.002, 0.011)]. The mediating effect difference between the high and low self-esteem conditions was −0.008, and the 95% CI was (−0.015, −0.002), excluding 0, thus revealing a significant difference in the mediating effect. That is, the mediating effect of job significance between received task interdependence and proactive work behavior was moderated by self-esteem. Hence, H_4b_ is supported by the data.

## 5. Discussion

Through the literature review, we found only few studies that distinguished the two dimensions of task interdependence according to the work flow direction. Most of these studies also took task interdependence as a whole construct. In reality, the different dimensions of task interdependence depending on the work flow direction vary in their impact on the attitude and behavior of employees. Our study revealed the effects of initiated task interdependence and received task interdependence (two dimensions of task interdependence) on employee proactive work behavior, and tested the hypotheses with first-hand data. The empirical findings are summarized as follows.

First, different dimensions of task interdependence (initiated task interdependence and received task interdependence) have varying effects on employee proactive work behavior. Initiated task interdependence has a positive impact on employee proactive work behavior, whereas received task interdependence has no significant effect on proactive work behavior.

Second, task significance mediates the relationship between initiated task interdependence and proactive work behavior.

Third, regardless of employees’ self-esteem being high or low, initiated task interdependence can improve employees’ perception of the significance of their work, indicating that the work feature of initiated task interdependence itself has a strong impact on employees’ perception of task significance. For individuals with high self-esteem, the relationship between received task interdependence and task significance is not significant. For individuals with low self-esteem, received task interdependence positively affects their perception of task significance. This is consistent with our expectation that self-esteem moderates the relationship between received task interdependence and employees’ perception of task significance.

Fourth, self-esteem moderates the mediating effect of task significance between received task interdependence and proactive work behavior. For individuals with high self-esteem, the mediating effect of task significance is not significant. For individuals with low self-esteem, task significance has a significant mediating effect. That is, self-esteem moderates the mediating effect of task significance between received task interdependence and proactive work behavior.

### 5.1. Theoretical contribution

We found a lack of research on the impact of task interdependence on proactive work behavior. Wu et al. (2018) studied the predictive effect of the interaction between task interdependence and individuals’ cooperative tendency on proactive work behavior, and introduced the influence of task interdependence on proactive work behavior. However, the aforementioned study did not consider the difference in the effects of various dimensions of task interdependence, depending on the work flow direction (initiated task interdependence and received task interdependence), on proactive work behavior. Most scholars regard task interdependence as a whole construct. The theoretical contribution of this paper is that it distinguished two different dimensions of task interdependence and empirically examined the differentiated impacts of the job characteristics of different work flows on employee proactive work behavior. The results revealed that initiated task interdependence can significantly increase employee proactive work behavior, whereas the characteristics of received task interdependence have no significant relationship with employee proactive work behavior.

Second, this study extends the theory of task interdependence and employee proactive behavior by demonstrating that the relationship is mediated by task significance. This finding broadens existing knowledge in two ways. First, it expands our understanding of how task interdependence may influence employee proactive behavior, which answers the call for research that focuses on a wider range of job characteristics and investigates their impact on employee proactive work behavior and the underlying mechanisms (Grant and Ashford, 2008). Otherwise, Wu et al. (2018) studied the moderating effect of task interdependence in the association between interdependent self-construction and team-oriented proactive behaviors. The mediating effect of task significance further enriches the current understanding of the role of task interdependence in fostering employee proactive behavior.

Third, this study further explored the moderating role of self-esteem in the relationship between initiated task interdependence and received task interdependence on employee proactive work behavior. For individuals with high self-esteem, the relationship between received task interdependence and job importance is not significant. For individuals with low self-esteem, received task interdependence positively affects employees’ task significance. Individuals with high self-esteem tend to be more confident in their capability, excellence, and success (Coopersmith, 1967), and may be more inclined to initiate tasks that affect others’ work, rather than merely accepting tasks that are influenced by others’ work. Thus, for individuals with high self-esteem, initiating tasks brings a greater sense of task importance than accepting them. In contrast, individuals with low self-esteem do not have confidence in their abilities and success, and experience no difference in the sense of work importance whether they accept or initiate tasks.

### 5.2. Managerial implications

The managerial implications of this study are discussed below:

First, the study’s findings serve as a reference for enterprises to arrange work tasks more reasonably. In traditional enterprises, employees mostly accept work arrangements from superiors, which does not help motivate employee proactive work behaviors. Enterprises can encourage employees to take the initiative according to the development direction of the company, initiate some work tasks, and take the lead to complete the work to promote their proactive work behaviors.

Second, different employees have different characteristics. For example, the level of self-esteem of employees in enterprises varies. Individuals with high self-esteem may perform better in initiated task-oriented positions but feel that their abilities are not appreciated in received task-oriented ones. Meanwhile, individuals with low self-esteem may perform better in received task -oriented positions. Therefore, employers need to consider the differences between individuals when allocating work, promote the advantages, and avoid the weaknesses.

Third, enterprises should be a community of persons who look to the common good (Melé, 2009, 2012), rather than focusing only on job design and becoming a passive profit-making entity. That is, enterprises should move beyond internal job design and establish a community from multiple perspectives. For example, realistic Personalism can be integrated into virtue-based business ethics by focusing on the Personalized Principle (emphasizing human dignity, self-esteem, and virtues) and the Common Good Principle (promoting conditions for the flourishing of all people within a community) (Melé, 2009).

### 5.3. Limitations and directions for future research

Despite the contributions mentioned above, our study is not without its limitations. First, all scales of the sample were filled in by the same employees from the same company; thus, this study has the problem of homologous variance. However, when conducting the Harman single factor test in SPSS (Statistical Product and Service Solutions), more than one factor was extracted, and the contribution rate of the first factor did not exceed 40%, such that the next statistical analysis could be carried out. Future studies should expand the sample to include different types of enterprises to enhance the external effect of the research conclusions.

Second, Future research can further investigate the effects of initiated task interdependence and received task interdependence on other employee behaviors and attitudes. Task interdependence is a particularly important feature of work in today’s economy and society, deserving greater attention. As Parker and Collins (2010) stated in their review of proactive work behavior, there are two major trends in the current organizational reform, one of which is that the degree of work interdependence within an organization is continuously increasing. More jobs require collaboration among colleagues. Therefore, further research on the influence of initiated task interdependence and received task interdependence on employees’ behavior and attitude can better guide practical work.

Third, this study only tested the moderating effect of self-esteem while controlling for gender, education, and position. However, there may be other potential variables that can also impact employee proactive behavior. Future research can consider controlling for additional factors, such as empathy (Zaki, 2014, 2020; Herrera et al., 2018; Luis et al., 2023) and the leader-member exchange relationship (Fein and Tziner, 2021). Examining the influence of task interdependence on employee proactive behavior while controlling for a more comprehensive set of variables can lead to more robust findings.

Finally, future research can take virtue ethics as a context in which job design humanizes the workplace. Job design is not an end, and its purpose is to make the people working the job feel dignity (Sison et al., 2016), self-esteem, and efficiency. Work interdependence should be effective when employees have positive, mature characteristics (Hartman, 1998, 2006). For example, despite the interdependence of two employees’ work, if the one initiating the task refuses to cooperate and help the other, who is in the downstream of the workflow, initiating the task cannot have affect the work of others. Therefore, great attention should be paid to connect the importance of virtues in production and interdependent tasks in future research.

## Data availability statement

The raw data supporting the conclusions of this article will be made available by the authors, without undue reservation.

## Author contributions

TY: conceptualization, investigation, and writing. YZ: conceptualization, methodology, data curation, and formal analysis. ZZ: resource and supervision. All authors contributed to the article and approved the submitted version.
